# GMP-Grade Manufacturing and Quality Control of a Non-Virally Engineered Advanced Therapy Medicinal Product for Personalized Treatment of Age-Related Macular Degeneration

**DOI:** 10.3390/biomedicines10112777

**Published:** 2022-11-01

**Authors:** Martina Kropp, Nina Harmening, Thais Bascuas, Sandra Johnen, Eline De Clerck, Verónica Fernández, Mattia Ronchetti, Ruggero Cadossi, Cristina Zanini, Daniel Scherman, Zoltán Ivics, Corinne Marie, Zsuzsanna Izsvák, Gabriele Thumann

**Affiliations:** 1 Group of Experimental Ophthalmology, University of Geneva, 1205 Geneva, Switzerland; 2 Department of Ophthalmology, University Hospitals of Geneva, 1205 Geneva, Switzerland; 3 Department of Ophthalmology, University Hospital RWTH Aachen, 52074 Aachen, Germany; 4 3P Biopharmaceuticals SL, 31110 Noain, Spain; 5 BVI c/o Optikon 2000 S.p.A., 00138 Rome, Italy; 6 IGEA S.p.A., 41012 Carpi, Italy; 7 BioAir S.p.A., 20016 Pero, Italy; 8 CNRS, Inserm, UTCBS, Université Paris Cité, F-75006 Paris, France; 9 Division of Medical Biotechnology, Paul-Ehrlich-Institute, 63225 Langen, Germany; 10 Chimie ParisTech, PSL Research University, F-75005 Paris, France; 11 Max Delbrück Center for Molecular Medicine in the Helmholtz Association, 13125 Berlin, Germany

**Keywords:** personalized medicine, autologous, regenerative medicine, cell product, cell therapy, Advanced Therapy Medicinal Product (ATMP), Good Manufacturing Practice (GMP), quality control, non-viral gene therapy, age-related macular degeneration (AMD), iris pigment epithelial cells (IPE)

## Abstract

The introduction of new therapeutics requires validation of Good Manufacturing Practice (GMP)-grade manufacturing including suitable quality controls. This is challenging for Advanced Therapy Medicinal Products (ATMP) with personalized batches. We have developed a person-alized, cell-based gene therapy to treat age-related macular degeneration and established a vali-dation strategy of the GMP-grade manufacture for the ATMP; manufacturing and quality control were challenging due to a low cell number, batch-to-batch variability and short production duration. Instead of patient iris pigment epithelial cells, human donor tissue was used to produce the transfected cell product (“tIPE”). We implemented an extended validation of 104 tIPE productions. Procedure, operators and devices have been validated and qualified by determining cell number, viability, extracellular DNA, sterility, duration, temperature and volume. Transfected autologous cells were transplanted to rabbits verifying feasibility of the treatment. A container has been engineered to ensure a safe transport from the production to the surgery site. Criteria for successful validation and qualification were based on tIPE’s Critical Quality Attributes and Process Parameters, its manufacture and release criteria. The validated process and qualified operators are essential to bring the ATMP into clinic and offer a general strategy for the transfer to other manufacture centers and personalized ATMPs.

## 1. Introduction

Gene therapy, cell therapy, and tissue engineering, known as ”advanced therapies” are the new frontiers for personalized treatments for currently untreatable diseases [1,2,3,4,5,6]. Respective Advanced Therapy Medicinal Products (ATMPs) are biopharmaceuticals, whose development is associated with novel challenges for production, quality control (QC), transport, storage and application [7,8,9]. Even when ATMPs are often produced for one patient [7,8,10], they must be produced and controlled under Good Manufacturing Practice (GMP) compliant practices and pharmaceutical conditions [11], which comprises standardized and validated manufacture and QC performed by qualified personnel using qualified devices, under rigorous hygienic conditions. Each step must be carefully documented to ensure the transparency and traceability. QC should ensure comparable, batch-independent efficiency and safety as defined by respective international regulations [11,12,13,14], which requires testing a small sample of raw, starting materials and of the end product before the release of a batch of the ATMP. However, for personalized, autologous, cell-based ATMPs, often small volume and time-sensitive application limit the time available for QC [7,8,10]. To date, an individual risk-based approach is required for every product [15], which hampers progress in the field. A general approach applicable to personalized ATMPs of small volume and short production time, would promote scientific progress and bring novel therapies into clinical routine more rapidly.

We are developing a non-viral cell-based gene therapy approach to treat neovascular age-related macular degeneration (nvAMD), which is the major cause of blindness in the elderly population of industrialized countries [14]. nvAMD is mainly driven by an imbalance of angiogenic and anti-angiogenic factors favoring an overexpression of the angiogenic vascular endothelial growth factor (VEGF) and including Choroidal Neovascularization (CNV), which requires frequent, often monthly life-long intraocular injection of inhibitors of VEGF [15,16]. To develop a once-for-life treatment for nvAMD, our laboratory, the group of Experimental Ophthalmology directed by Prof. Thumann, University of Geneva, has shown that primary human and animal iris pigment epithelial (IPE) cells are efficiently transfected ex vivo with the *Pigment Epithelium-Derived Factor* (*PEDF)* gene using the *Sleeping Beauty* (*SB*) transposon system, which integrates the gene into the host genome [17,18,19,20]. For treatment of nvAMD in humans by transplantation of PEDF-transfected IPE cells, it is required that (a) the IPE cells are autologous to avoid immune rejection, (b) the cells are not pre-cultured to avoid microorganism contamination, (c) the possibility that extracellular proteins from the culture medium contaminate the autologous cells is prevented, and (d) the loss of cells’ phenotype during culture is avoided (fibroblastic phenotype). Thus, the clinical approach comprises obtaining an iris biopsy from the nvAMD patient, isolating and transfecting IPE cells with the human *PEDF* and *SB* genes, consisting of the transposon and the helper transposase, and transplanting the final product (“tIPE”) subretinally into the same patient within 60 min during a single surgical session at a reasonable cost. According to a clinical trial performed by Lappas et al. in which non-transfected IPE cells were transplanted to AMD patients, a low number of cells (10,000–20,000) in the final product is expected [21] This is not compatible with the collection of a sample from the tIPE for QC. Additionally, the transport of tIPE is a challenge, since the biological product needs to be transported from the clean room (or isolator) to the surgery room for transplantation at GMP-compliant conditions (i.e., triple packaging) without hampering its quality [22]. To minimize manipulation steps, tIPE is directly loaded into a sterile microsyringe (Hamilton), ready for injection. The transport of tIPE inside a syringe towards the surgery room is another critical step before administration.

Here, we report the validation procedure according to GMP [11] for the ATMP tIPE, including performance qualification (PQ) of devices, qualification of personnel, and propose a comprehensive process for the release of cell-based personalized therapeutic medicinal products, as well as the development of a bioconfined container to ensure the safe transport of the ATMP from the production to the surgery site [23,24]. To confirm feasibility of the treatment procedure including biopsy harvest, cell isolation, ATMP production and transplantation within 60 min, a proof-of-principle study in rabbits has been performed; the rabbit has been chosen as model due to comparable size of the eye.

## 2. Materials and Methods

### 2.1. Transfection Consumables and Equipment

During the TargetAMD project (www.targetamd.eu, accessed on 11 January 2012), research and GMP grade (produced by AmBTU, Amsterdam, Netherlands [25,26,27]) plasmid mixtures, which contained 226 ng pFAR4-CMV-SB100x transposase and 3624 ng pFAR4-ITRs-CMV-PEDF-BGH transposon plasmids were suspended in 25 µL of 3P.14 electroporation buffer developed by 3P Biopharmaceuticals (Navarra, Spain) [26]. The customized Cliniporator^TM^ and electroporation cuvettes, developed for the electroporation of small volume cell samples by partner IGEA S.p.A. (Carpi, Modena, Italy), were used to transfect the IPE cells with the plasmid mixture [26,28].

### 2.2. Rabbit Proof-of-Principle (PoP) Study

#### 2.2.1. Animals and Iridectomy

For the in vivo validation of iridectomy, cell isolation, transfection and transplantation, four normal Chinchilla Bastard rabbits bred in our animal facility (University of Geneva, Geneva, Switzerland) had access to food and water *ad libitum* and were housed in groups whenever possible, under controlled humidity and temperature on a 12 h light/dark cycle. Iridectomy was performed under general anesthesia using Ketalar (Pfizer, Zurich, Switzerland) (35 mg/kg) and Domitor (Orion Pharma, Espoo, Finland) (0.5 mg/kg) or Rompun (Bayer, Leverkusen, Germany) (3 mg/kg) diluted in NaCl injected intra-muscularly; anesthesia was maintained by injecting a half dose every 30 min if necessary. After sedation, the pupils were dilated using Tropicamide eye drops (0.5%, Alloga SA, Burgdorf, Switzerland), eyes were anesthetized by a drop of Tetracaine (1% 0.4 mL; AMEDIS-UE AG, Unterentfelden, Switzerland), and drying of the cornea was avoided by regularly administering humidifying Methocel gel (2%, Amedis-UE AG, Unterentfelden, Switzerland).

**Figure 1:**
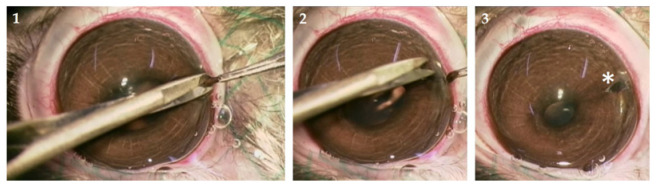
Iris biopsy. A small incision was made in the cornea, iris was grasped with small forceps, pulled from the anterior chamber (panel 1), and a 1 mm × 2 mm biopsy cut (panel 2). The cut in the cornea sealed without suturing; the asterisk (* panel 3) marks the iridectomy site.

To obtain iris biopsies, a 2–4 mm cut was made close to the limbus using an ophthalmic knife (Mani, Tochigi, Japan) in the right eye under a surgical microscope (Zeiss, Jena, Germany). The iris was grasped with Colibri forceps and a 1–2 mm section was cut with micro-scissors; the iris spontaneously repositioned in the anterior chamber of the eye and the cut healed without suturing (Figure 1). After a follow-up of 7 days (d) or 90 d, the animals were sacrificed by an overdose of Thiopental (Ospedalia AG, Hünenberg, Switzerland) (500 mg/8 mL NaCl) injected intravenously under general anesthesia.

#### 2.2.2. Rabbit tIPE Production

To isolate IPE cells, biopsies were transported to a laminar flow hood and placed in a 10 cm petri dish containing 500 µL of 0.05% trypsin/0.5 mM EDTA; after 75 min incubation at 37 °C, the trypsin solution was removed and 500 µL DMEM/Hams 12 medium containing 10% fetal bovine serum (FBS) were added to stop trypsinization. IPE cells were gently scraped from the stroma using a round ophthalmic knife (Mani, Tochigi, Japan) and centrifuged at 120 g for 10 min at room temperature (RT); after aspiration of the supernatant, the pellet was suspended in 15 µL of 3P.14 buffer and transferred to a 1.5 mL tube to which were added 5 µL of the plasmid mixture [25]. Cells were transfected by electroporation using the Cliniporator^TM^ (1 pulse, 200 V, 5 ms) as described [26]. After transfection, the 20 µL of transfected IPE cells were transferred to a 1.5 mL tube and the electroporation cuvette washed two times with 40 µL BSS Plus (Balanced Salt Solution Plus, Alcon, Zug, Switzerland), which was added to the 20 µL of cells. After taking 10 µL of the suspension for cell counting, it was centrifuged at RT for 10 min at 120 g to remove remnant extracellular DNA (including DNA released by damaged cells and plasmid DNA that did not enter cells). The cell pellet was resuspended in a concentration of 20,000 cells/30 µL of BSS Plus and drawn into a 100 µL gastight Hamilton syringe (Hamilton, Bonaduz, Switzerland) connected to a 35 cm long Silkflex tubing with an attached 35G blunt needle for subretinal injection (“RPE-kit”, WPI, Friedberg, Germany).

#### 2.2.3. Transplantation of tIPE in Rabbits

One or three trokars (TOTALPlus 23Ga VITRECTOMY Pak, Alcon, Zug Switzerland) were inserted into the sclera of the right eye proximal to the limbus on either side of the globe, one to serve as the light source and the second and third to serve as the injection port. The blunt 35G needle attached to the Silkflex tubing was carefully pushed into the injection port trokar, positioned just below the bundle of nerve fibers close to the papilla and 20,000 cells in the Hamilton syringe suspended in 30 µL BSS Plus were injected subretinally. Then, the trokars were removed; the lacerations made by the trokars healed without suturing. Unlike in humans, vitrectomy in rabbits is not required to transplant cells subretinally. Rabbits were followed for 7 d or 90 d. The left eye served as untreated control.

#### 2.2.4. Intraocular Pressure (IOP) and Fundoscopy

The IOP was measured in the awake animal before, 7 d or 90 d post-surgery using the Tonovet (Icare, Vantaa, Finland) tonometer to exclude harmful alterations. A single-use probe was pressed slightly onto the cornea five times and the re-bound measured; the mean of the five measurements was recorded as the IOP. A video of the fundus and the injection site of both eyes was examined before, 7 d or 90 d post-surgery using a surgical microscope (Zeiss, Jena, Germany) and a 1.0X Volk^®^1 Single-Use Flat Lense (Volk, Cleveland, OH, USA). A drop of Methocel on the surface was used to prevent drying and improving image quality.

#### 2.2.5. Examination and Dissection of Rabbit Organs

Rabbits were inspected *post-mortem* to determine whether transplantation of PEDF-transfected cells had any macroscopical site effect. The organs inspected in situ visually were: the tear glands, salivary glands, harderian gland, pituitary gland, tongue, esophagus, thyroid and parathyroid glands, thymus, ribs, aorta, gallbladder, stomach, gut, bladder, prostate, seminal vesicle, pancreas, adrenal glands, muscles, peripheral nerves, bone marrow, spinal cord, and lymph nodes. The organs dissected after visual examination were: the cerebrum, cerebellum, optic chiasma, optic nerve, heart, lung, liver, spleen, kidney, and testes/ovaries. The skull was opened with a bone tongs. The trunk was opened with an incision from throat to abdomen to inspect and dissect listed organs. The muscles of one thigh were exposed and a peripheral nerve was dissected free and both were visually examined. Bone marrow was collected after the thigh bone was ruptured. The spinal cord was dissected and visible lymph nodes were inspected. If an organ appeared visually abnormal, a biopsy was sent for analysis to a veterinarian pathologist from IDEXX B.V. (Hoofddorp, The Netherlands).

### 2.3. Validation Run

#### 2.3.1. Human tIPE Production

The tIPE (ATMP) manufacture was done by one operator (working under a laminar flow hood) and one assistant (working outside of the laminar flow hood) in the laboratories of the clean room facility of the University Hospitals of Geneva (HUG), Switzerland (Laboratoires de Transplantation & Thérapie Cellulaire, LTTC). During training and validation, human iris biopsies from 42 eyes (2–8 d *post-mortem*) from 28 donors purchased from the Lions Gift of Sight Eye Bank (Saint Paul, MN, USA) were used as starting material.

To obtain iris biopsies, after donor eyes were disinfected in betadine (Mundipharma, Frankfurt, Germany) for 90 s, a simulated iridectomy under a laminar flow hood in a cell laboratory with a similar distance to the LTTC as has the surgery site in the planned clinical trial, was done. A small incision was made close to the limbus with a lancet-formed ophthalmic knife (Mani, Tochigi, Japan), the iris was pulled slightly and a small piece of the iris was cut using micro-scissors and placed into a 1.5 mL tube containing 20 µL 3P.14 electroporation buffer. Six biopsies were taken from each eye with 4 iris samples used to produce tIPE (i01-4) and two iris control samples (Co-1 and Co-2), to isolate cells for counting and determine cell viability. Iris biopsies for tIPE production were brought to the LTTC, where the operator placed the biopsy in a 6 cm petri dish and added 17 µL of 3P.14 buffer. Using a lancet-shaped ophthalmic knife (Mani, Tochigi, Japan) to hold the biopsy, the IPE cells were carefully scraped from the stroma using a round ophthalmic knife (Mani, Tochigi, Japan). Fifteen microliters of the cell suspension were transferred into a 1.5 mL tube to which were added 5 µL of plasmid mixture and cell clumps disrupted by up and down pipetting 2–3 times. The 20 µL mixture was transferred into the Cliniporator^TM^ microcuvette and electroporated (1 pulse, 200 V, 5 ms). The 20 µL of PEDF-transfected cell suspension were transferred to a 1.5 mL tube, the cuvette washed twice with 40 µL BSS Plus and the washes added to the cell suspension. Ten microliters of the cell suspension were used undiluted for cell counting in a C-Chip Neubauer Chamber (NanoEnTek, Seoul, Korea) under a microscope (Nikon, Tokyo, Japan) by the assistant; the remaining 90 µL were centrifuged at 120 g for 10 min at RT. The supernatant from the centrifugation was removed and used for QC; the cell pellet was resuspended in 50 µL BSS Plus, i.e., tIPE. After removing a sample of 1000 cells, as determined from the cell count before centrifugation, to assess viability (see Section 2.2.5), remaining tIPE was transferred into a gastight 100 µL Hamilton syringe without producing air bubbles; the final volume loaded into the syringe was noted.

#### 2.3.2. Bioconfined Syringe Container

The ATMP bioconfined container was constructed by BioAir S.P.A. (Pero, Italy) following the general scheme for the development of bioconfined systems for the transfer and handling of ATMPs, which comprises the steps “definition”, “design”, “prototype” and “validation” [29,30]. The project details were defined with the input of the tIPE operator (Step 1), considering (a) the size of the syringe, (b) needle connection to the syringe immediately before tIPE transplantation, (c) volume of the final product, (d) possible need of a filter for gas exchange, (e) maintaining sterility during transport, (f) need to have an autoclavable case and a sterile disposable interior, (g) need to prevent movement of syringe plunger during transport to avoid tIPE dispersion, (h) number of syringes transported per month, and (i) cost of ATMP. The prototype design (Step 2) considered material, methodology and engineering [31]. For the outer parts, an autoclavable material was chosen, whereas for the inner part, a sterile and disposable material was selected. The prototype was produced using 3D injection printing and molding (Step 3). In Step 4, the producer tested the suitability and usability of the prototype including loading the container, fitting the syringe in the internal chamber, sealing, transporting, and general handling.

#### 2.3.3. Cell Viability

Cell viability was determined using the CytTox Glo^®^ assay (Promega, Madison, WI, USA) following the manufacturer’s instructions. Briefly, after thawing and preparation of substrate and assay buffer, 1000 cells from the 4 tIPE and 2 controls from each eye were dispensed into 6 wells of a 96-well plate to which was added BSS Plus to 200 µL. After adding 50 µL of assay buffer, samples were incubated at RT for 15 min and luminescence (L1) determined. Then, 50 µL of the lysis reagent were added to the wells and after 15 min incubation at RT, luminescence (L2) was determined. Percentage of viable cells is the difference between L2 and L1. To construct a standard curve, a confluent well of a 6-well plate of ARPE-19 cells (156 to 5000 cells in a 1:2 dilution series) was trypsinized for 7 min at 37 °C by adding to the well 200 µL of 0.05% Trypsin/0.5 mM EDTA. Trypsinization was stopped by adding 400 µL DMEM/Ham’s 12 medium supplemented with 10% FBS; the cell suspension was centrifuged at 700 rpm for 7 min at RT. The supernatant was aspirated and the cell pellet resuspended in 400 µL DMEM/Ham’s 12 and the assay performed as described above.

#### 2.3.4. Contaminating Remnant Extracellular DNA

To verify the purity of tIPE, the DNA content in the centrifugation supernatant (in the following “remnant extracellular DNA”) was determined using the Qubit^®^ dsDNA HS Assay (ThermoFisher Scientific, Waltham, MA, USA) according to manufacturer’s instructions (detection limit: 0–1000 ng dsDNA). Three microliters of the supernatant were mixed with 597 µL of the working solution provided by the manufacturer; 200 µL of the assay solution were pipetted in a 96-well plate and fluorescence read (excitation: 485 nm; emission: 520 nm) using the Omega spectrophotometer (BMG Labtech, Ortenberg, Germany). Remaining plasmid mixture solution from tIPE manufacture (one vial contained 25 µL but used were only 5 µL) was used to prepare a standard curve from 0.6 to 9.63 ng/µL in a 1:2 dilution series.

**Figure 2:**
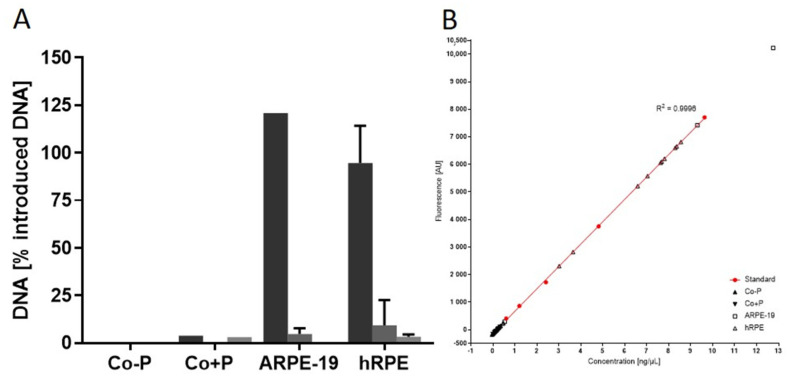
Recovery of DNA in the supernatant of centrifuged transfected cells. (**A**) Cultured hRPE and ARPE-19 cells, transfected with the PEDF-plasmid construct were centrifuged 3 times and the DNA in the supernatant determined. Two controls were analyzed: hRPE cells not electroporated nor mixed with DNA (Co-P), hRPE cells electroporated without addition of DNA (Co+P) cells. Only after electroporation DNA was detected in the supernatant. In both groups of transfected cells, the majority of introduced DNA was recovered after 1 centrifugation (ARPE-19: 120.82%; hRPE: 94.6 ± 19.6%). After the second and third centrifugations, only small amounts of DNA were detected and only in some samples. Second centrifugation: ARPE-19 = 4.8 ± 3.0% (n = 2); hRPE = 9.3 ± 13.2% (n = 7). Third centrifugation: hRPE = 3.4 ± 1.1% (n = 3). (**B**) Standard curve for DNA quantification. n = 2 (Co-P, Co+P, ARPE-19) and n = 9 (hRPE).

The assay has been established in cultured hRPE and ARPE-19 cells (Figure 2). From Gillooly et al. data on DNA content of multiple cells [32], the DNA content of 10,000 cells has been estimated to 52 ng. In the transfection of cells with the *PEDF* gene, we delivered 770 ng of plasmid DNA. Only 0.7 ng of the added DNA is necessary to transfect 10,000 cells with 1 *PEDF* gene-copy; thus, the majority of the 770 ng added DNA should be retrieved in the supernatant after centrifugation of transfected cells. To verify the efficacy of centrifugation in extracellular remnant DNA removal, 10,000 ARPE-19 and primary human RPE cells were transfected with the PEDF-plasmid construct. Non-electroporated cells (Co-P) and electroporated cells (Co+P), both without PEDF-plasmid construct added, served as controls. Figure 2 shows that no nucleic acid was measured in the supernatant of Co-P cells whereas low but detectable amount of DNA was present in the Co+P supernatant, suggesting that electroporation has damaged some cells, releasing DNA and explaining DNA in the supernatant of tIPE exceeding 100%. In all transfected cells, the majority of the added DNA was retrieved in the supernatant of the first centrifugation (ARPE-19 cells: 120.82%; hRPE cells: 94.6 ± 19.6%). After the second and third centrifugations, DNA in the supernatants was not detected or only small amounts of less than 10 ng DNA were found. In one ARPE-19 cell sample, DNA in the supernatant was remarkably high after the first centrifugation with a value out of the linear range of the standard curve. The value has been excluded from the analysis (“invalid measurement”). Comparable values measured in the validation batches were similarly excluded.

#### 2.3.5. Product Volume and Appearance

tIPE was transferred into a sterile 100 µL microsyringe using a 23 G needle (Beckton & Dickinson, Franklin Lakes, NJ, USA) comprising the transfected cells suspended in 50 µL BSS Plus, a common intraocular irrigating solution. The actual volume in the syringe was documented, since the sample removed for cell viability measurement, cell clumps, needle’s dead volume and variations in operator’s performance could impact the volume dispensed into the syringe. The cell suspension in the syringe was inspected for the absence of air bubbles and contaminating particulate matter; presence of air bubbles and particulate matter would require refilling of the syringe.

#### 2.3.6. Production, Transport Time and Temperature

Transport time was recorded as close as possible from the time of biopsy harvest to the start of cell isolation (“T1”) and the time (“T2”) from tIPE production completion to arrival into the cell laboratory for validation purpose (see Section 2.3). T1 was recorded a few minutes longer than the exact value, since the operator stopped the timer after he transferred the biopsy to the production laboratory and returned to the locker room of the LTTC (the platform comprises two locker rooms, four laboratories, one QC room, and two storage rooms); T2 was recorded a few minutes shorter than the exact value, since the operator started the timer after receiving the tIPE and left the locker room. To prevent cell deterioration, the temperature of tIPE during transport should be lower than 37 °C to slow down metabolism but above freezing to avoid cell damage; we defined the allowed temperature range from 4° to 25 °C controlled by cooling the transport box with cooling packs. Temperature was documented by a calibrated thermometer for transport of the biopsy to the LTTC and the transport of tIPE to the cell laboratory.

#### 2.3.7. Endotoxin

Gram negatvie bacterial endotoxin levels were determined in the supernatant after plasmid-transfected cells were centrifuged using the Endosafe Nextgen PTS assay (Charles River, Wilmington, MA, USA) with cartridges of 1.0–0.01 EU/mL sensitivity according to the manufacturer’s instructions. Five microliters of supernatant were diluted 1:100 with LAL (Limulus amebocyte lysate) Reagent and 25 µL of the suspension were pipetted into each of the four wells of the cartridge by the assistant. A value of <0.2 EU/mL was conforming to tIPE release criteria. In case of an invalid value (not non-conforming, but unusable due to assay performance errors), the measurement was repeated; however, after two invalid measurements or a non-conforming value with an endotoxin level greater than 0.2 EU/mL, the QC result was documented as non-conforming. The Endosafe Nextgen PTS documents the endotoxin value as well as temperature, sample reaction time, spike reaction time and spike recovery, which allows the detection of invalid measurements due to technical difficulties.

#### 2.3.8. Microbiological and Environmental Controls

**Figure 3:**
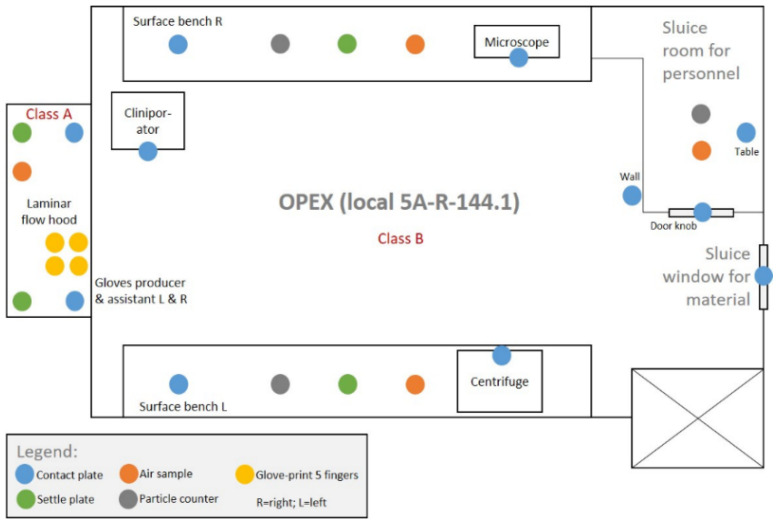
Environmental monitoring during production. The figure illustrates the laboratory room of the LTTC with its critical compartments and devices. The airlock for the entry of personnel (class C) is tested for particles, microbes in the air and on doorknob and table; the sluice window for the entry of material is tested with a contact plate. In the class B laboratory, all surfaces and one wall are tested for particle and microbiological matter using the air-sampler, and for living organisms using the settle and contact plates; all devices (centrifuge, microscope, Cliniporator^TM^) are tested for microorganisms by contact plates. The interior of the Class A laminar flow hood and personnel gloves are tested for contaminations. A total of 26 sites are tested, i.e., 4 air samples, 3 particle counts, 11 contact plates, 4 settle plates, 4 glove prints.

Additional to quarterly performed environmental controls, for every production the environment, the starting material, and the product were tested for contamination by aerobic, anaerobic bacteria, fungi, and non-viable particulate matter. The starting material and tIPE were tested by culturing in BHI (Brain Heart Infusion) medium on agar plates until a positive result (bacterial growth in the sample tested) or for two weeks. After cell isolation, the iris biopsy stroma (considering the biopsy as starting material) was tested for potential contaminations and 75 µL of the centrifugation supernatant were used to confirm sterility of tIPE. Figure 3 illustrates the locations in the laboratory where samples were collected and analyzed for contamination by culturing in BHI medium until a positive result was evident or for two weeks. One air sample was taken in the sluice room, one on each of the laboratory benches and one in the laminar hood using an air sampler (Biomérieux, Petit Lancy, Switzerland) collecting 1000 L/~10 min. Live microorganisms were determined by settle plates (Merck, Kenilworth, NJ, USA) one on either side of the workspace under the laminar hood and one on either laboratory bench. Particle contamination in the air was determined using a particle counter (Bakrona, Basel, Switzerland) in the air of the laboratory and in the sluice room by collecting 500 L/~10 min (production rooms) or 150 L/~10 min (sluice room). Contact plates for environmental monitoring (Merck, Kenilworth, NJ, USA) were used to monitor contamination in several areas as indicated in Figure 3. Both, the assistant and the operator tested their right and left gloves by a 5-finger-touch on one contact plate for each glove (Merck, Kenilworth, NJ, USA).

### 2.4. Statistics

Descriptive statistical analysis was performed for all data (mean, SD, SEM, min, max, range). A D’Agostino & Pearson normality test identified parametric and non-parametric data. Depending on the nature of the data, significant differences between groups were calculated using the unpaired parametric *t*-test (2 samples), ANOVA (≥3 samples), unpaired non-parametric Mann-Whitney (2 samples) or Kruskal-Wallis (≥3 samples) test. If not noted otherwise, data are shown as mean ± SD. All analyses were made using GraphPad Prism^®^ software version 9.3.1 (471) (GraphPad Software, LLC, San Diego, CA, USA).

## 3. Results

### 3.1. PoP Study—Validation Run in Rabbits

The study focused on the feasibility of manufacturing and transplantation of an autologous non-viral ATMP for personalized treatment of nvAMD performed in normal rabbits.

#### 3.1.1. Surgical Complications and Side Effects

Rabbits were used to verify feasibility of the treatment process. The small cut made in the cornea close to the limbus, was sufficient to obtain an iris biopsy of approximately 1 mm × 2 mm (Figure 1). The procedure was successful with operator-caused lens damage in two eyes; touching of the lens is not an anticipated risk in humans because the larger human eye with a proportionally smaller lens and by the procedure done by experienced surgeons. No adverse reactions were observed during the follow-up period (7 d and 90 d) (Table 1).

#### 3.1.2. Fundus and IOP

Theoretically, the surgery could affect the retina and the IOP and thus, were monitored by fundoscopy and tonometry. Fundus did not show pathological alterations or differences between the treated (right) and the contralateral (left) eye (Table 2). A small, not statistically significant decrease in IOP was observed during the first post-surgical week; 6.0 ± 1.4 mmHg in the treated eye vs. 9.8 ± 1.7 mmHg in the contralateral eye post-surgical at day 7. At day 90, IOP in the treated eyes was 8.0 ± 1.4 mmHg and 9.0 ± 1.4 mmHg in the contralateral eye. Base values were 11.5 ± 2.4 mmHg for the to-be-treated eye and 11.3 ± 2.2 mmHg in the contralateral eye.

#### 3.1.3. Organ Phenotype

To determine potential systemic side effects, organs’ morphology was evaluated. Organs were inspected visually and no abnormalities, defined as the absence of lesions, tumors, hemorrhages and signs of inflammation, i.e., edema or redness, were observed.

### 3.2. Bioconfined Syringe Container

To ensure sterility and viability of tIPE during transport from the manufacturing facility to the surgery site, a reusable transport box was manufactured comprising an outer opaque bottom shell and an outer transparent upper shell of temperature resistant, autoclavable plastic, and an inner syringe container fabricated of a non-porous soft plastic to secure the syringe in place preventing movement and fluid dispersal. A silicon gasket between the two outer shells results in an airtight seal when the two shells are fastened together with a clip at each end. The outer and inner container can be sterilized using gamma irradiation. For GMP-compliant use, it will be supplied with triple layer packaging to be opened in the controlled environment of the clean room. Throughout the whole transportation, the syringe will be firmly kept in a secure position and the piston cannot move accidentally, thus avoiding to expel the contents as long as the container (and the syringe inside) are kept in a horizontal position.

### 3.3. GMP-Grade Validation Run

#### 3.3.1. Framework—Purpose, Donors and Process

The validation run (ValRun) was designed to satisfy multiple requirements; the 143 tIPE productions performed were used to:

- Validate the process;
- Validate the cleaning procedure;
- Optimize the process under GMP-compliant conditions;
- Define release criteria;
- Collect sufficient in vitro data from human donor eyes to demonstrate quality of tIPE;
- Qualify the Cliniporator^TM^ (performance qualification, PQ);
- Train (carrier, assistant, operator) and qualify personnel (operator).

The validation was performed between 29 June and 1 November 2018 using a total of 42 eyes (Table 3). The team consisted of four experienced researchers, of which three were trained as tIPE operators, one team member was responsible for production (RP), a second was responsible for the equipment (RE), and a third was responsible for QC (RQ).

Parameters were recorded as conforming or non-conforming (“out of specification”, OOS, e.g., low cell number), which is different from being valid or invalid due to technical reasons (e.g., failed “Spike Recovery” in the endotoxin measurement). A complete production was defined as being compliant or non-compliant.

Validation benchmarks were defined as follows:

Conformity of release criteria: (a) ≥80 valid productions; (b) mean values must be conforming; (c) ≥75% of productions must be conforming; (d) at least the last 6 consecutive productions must be conforming.

Compliance of a production: (a) ≥80 valid productions; (b) mean values of individual parameters must be conforming; (c) ≥50% of the batches must be compliant in all parameters; (d) at least the last 6 consecutive productions must be compliant.

Personnel qualification: (a) ≥20 valid productions; (b) mean values of individual parameters must be conforming; (c) ≥50% of productions must be compliant.

**Figure 4:**
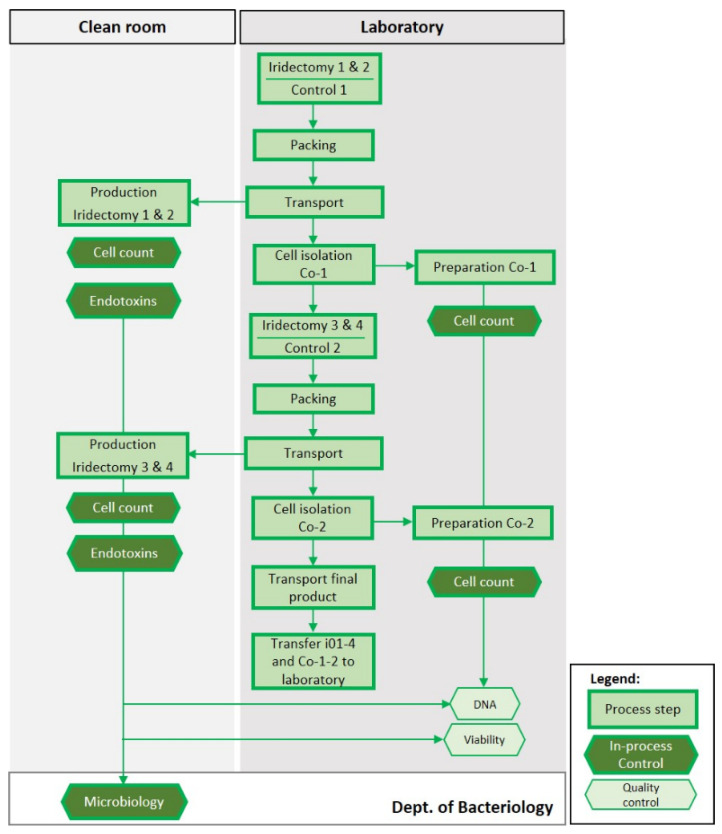
Flow chart of the ValRun. Iris i01–i04 and control biopsies (Co-1 and -2) were obtained from *post-mortem* human donor eyes; biopsies i01, i02, 103, and i04 were packaged and transported to the LTTC. The operator and the assistant manufactured tIPE 1 and 2 and separately tIPE 3 and tIPE 4, and prepared the samples for QC, which included cell count, endotoxin levels, microbiology, remnant extracellular DNA, and cell viability. In the laboratory, Co-1 and Co-2 were processed similar to i01-2 and i03-4 but under research laboratory conditions and without transfection. Stroma and supernatant of i01-i04 were submitted for microbiological analysis. tIPE1-tIPE4 were transported back to the laboratory, where also the remnant extracellular DNA and cell viability (also for Co-1 and -2) were analyzed.

The complete process is presented in Figure 4. The carrier prepared i01-2 and Co-1 in the laboratory and packaged the iris biopsies for transport. i01-2 were brought to the LTTC. Upon return to the laboratory, the carrier isolated the cells from Co-1, centrifuged the sample, aspirated the supernatant and resuspended the cells in 20 µL 3P.14 buffer (GMP-grade); 1000 cells were analyzed for cell viability. During the centrifugation step, a 10 µL aliquot of the isolated cells were counted in a Neubauer chamber. In the LTTC, the production team isolated and transfected the cells from biopsy i01 and 2 (as detailed in Section 2.3.1). Then, 5 µL of the centrifugation supernatant were used to determine the level of endotoxins. After termination of the production, tIPE was filled in the syringe ensuring the absence of air bubbles. The whole process should not take longer than 30 min. The carrier recorded duration and temperature of the transport and controlled the closure of the transport box by the integrity of a designated tape. The RQ controlled correct and readable labelling of tIPE and the completeness of the documentation. The production team kept 3 µL of the centrifugation supernatant to quantify remnant extracellular DNA; 1000 cells were analyzed for cell viability. The iris stroma and the remaining 70 µL from the supernatant of the centrifugation of the transfected cells were prepared for microbiological analysis. The process was repeated for i03-4 and Co-2 (biopsies were taken from the same donor eyes as for i01, i02 and Co-1). Viability of all products (i01-4) and controls (Co1-2) and the DNA concentration in the centrifugation supernatant were analyzed under laboratory conditions; stroma and supernatant, immersed in BHI medium, were analyzed for microbiological contamination as were all environmental controls by the Dept. of bacteriology, HUG.

#### 3.3.2. Process Optimization and Definition of Release Criteria

After an *interim* analysis, the first 39 productions were defined as “training phase” used to optimize the process, define release criteria and train personnel. This was followed by the subsequent 104 validation productions. Since microbiological analysis requires a minimum of 5 days, it is not possible to obtain microbiological results for the proposed treatment that requires cell transplantation within approximately 60 min from biopsy to transplantation. However, the microbiological analysis has been and will be necessary for therapeutic decisions in the unlikely case that an infection would occur post-transplantation. Table 4 details the specifications and QC performed during production and required for batch release. Apart from the QC reported above, all starting material was analyzed for completeness and validity. To deliver maximal data in the validation, it has been decided to continue the production under circumstances that may lead to a stop of the production in the clinical trial.

**Figure 5:**
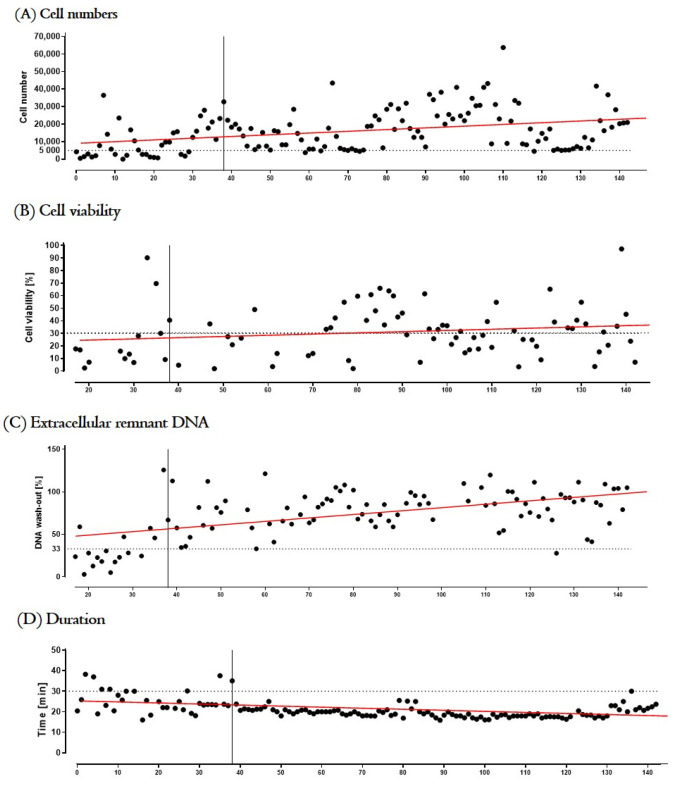
Effect of training on manufacturing of tIPE. Productions (n = 143) were analyzed to define the training necessary to manufacture a tIPE with optimal cell numbers isolated from a biopsy (**A**), cell viability (**B**), least contamination with remnant extracellular DNA (**C**), as well as time of manufacture of tIPE (**D**) routinely. The dashed lines present the lower limit of the release criteria (5000 cells, 33% remnant extracellular DNA, 30 min) or guidance level (30% viability). The vertical lines illustrate the end of the training phase and start of the ValRun (after 39 productions). (**A**) The number of isolated cells increased significantly over time even after the training period. (**B**) Cell viability increased over time, but the increase was not statistically significant. (**C**) Remnant extracellular DNA determined in the supernatants of tIPE improved significantly over time. (**D**) Manufacture duration was significantly shortened by training.

Figure 5 illustrates the effect of personnel training on “cell number”, “cell viability”, “remnant extracellular DNA” and “time of the manufacture of tIPE”; the mean values of productions 1–39 (training phase) and productions 40–143 (validation) were compared. The operators isolated a significant (*p* < 0.0001) higher number of cells per biopsy over time (10,487 ± 9798 cells vs. 17,882 ± 11,725 cells). Cell viability increased slightly but not significant (*p* = 0.1239) over time (25.56% ± 25.56% vs. 31.92 ± 19.04%). The remnant extracellular DNA concentration increased significantly (*p* < 0.0001) over time (35.53 ± 28.93% vs. 80.32 ± 21.31%). Similarly, the time of production was significantly (*p* < 0.0001) shortened by training (25.33 ± 5.72 min vs. 19.59 ± 2.39 min). The other release criteria were conforming from the first production on.

#### 3.3.3. Validation Run

Productions for validation and qualification were performed using donor eyes as specified in Table 3 Donors’ “age”, “time of death to cell isolation” and “gender” were not statistically different between the training and validation phases (age: *p* = 0.1706; time of death to cell isolation: *p* = 0.4733; sex: *p* = 0.7047).

**Figure 6:**
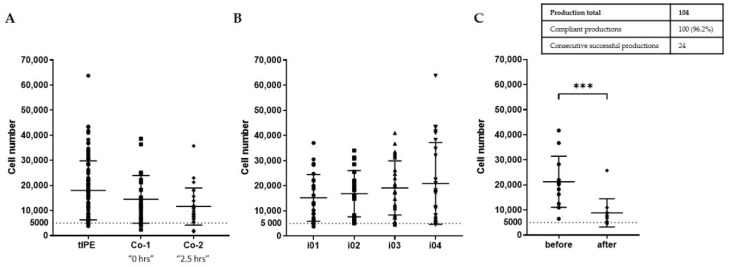
Number of cells isolated from one iris biposy. (**A**) The mean number of cells isolated in the clean room from all biopsies harvested for tIPE production was 17,882 ± 11,725. The number of cells cells isolated from the biopsies in the GMP-grade LTTC by operators was not statistically different from the number of cells isolated from the biopsies in research conditions by the carrier (*p* > 0.999). (**B**) The analysis of cell counts of the four iridectomies harvested per eye individually showed comparable cell numbers between the biopsy taken immediately after opening the eye (i01) and the last biopsy (i04) (*p* > 0.999). (**C**) To quantify cell loss by centrifugation and verify that tIPE contained the conforming number of cells, the cell number was determined in 12 iris biopsies before and after centrifugation. Cell number after centrifugation was significantly lower than before centrifugation (*p* = 0.0005, ***); however, the cell number was ≥5000 in 11 of 12 experiments. The dashed line marks the limit for product release.

*Cell number.* A sufficient number of cells in tIPE is crucial for successful transplant engraftment and levels of therapeutic protein expression. Previous in vivo experiments in rats have demonstrated that 5000 transplanted cells secrete a sufficient amount of PEDF to inhibit neovascularization [33]. During the ValRun, 17,882 ± 11,725 cells were isolated from biopsies (Figure 6). To investigate the effect of time between the first and last biopsy, we isolated cells from two control biopsies taken immediately after opening the eye (Co-1: 14,460 ± 9501 cells) and taken after the fourth iridectomy (Co-2: 11,605 ± 7412 cells) with an average 2.5 h between first and fourth biopsy. There was a decrease in number of cells isolated (Co-1 and Co-2); however, it was not statistically different (*p* > 0.999). On the other hand, the number of cells isolated from the four iridectomies showed a small but statistically not significant (*p* > 0.999) increase from i01 to i04 (i01: 15,183 ± 9261 cells; i02: 16,846 ± 9215 cells; i03: 19,111 ± 10,752 cells; i04: 20,913 ± 16,263 cells). Cells were counted before centrifugation despite the fact that centrifugation reduces cell number. To quantify the effect of centrifugation on cell number and verify that tIPEs contained a minimum of 5000 cells, cells were counted before and after centrifugation in 12 tIPE productions. Note the significant loss of cells, i.e., 21,271 ± 10,201 cells before centrifugation and 8833 ± 5628 cells after centrifugation. Even though the mean of the samples differed significantly (*p* = 0.0005, ***), the cell number was above the defined 5000 cells in 11 of the 12 tested productions. Of the 104 total productions, cell count was conforming in 100 (96.2%) cases, including the last 24 consecutive productions.

**Figure 7:**
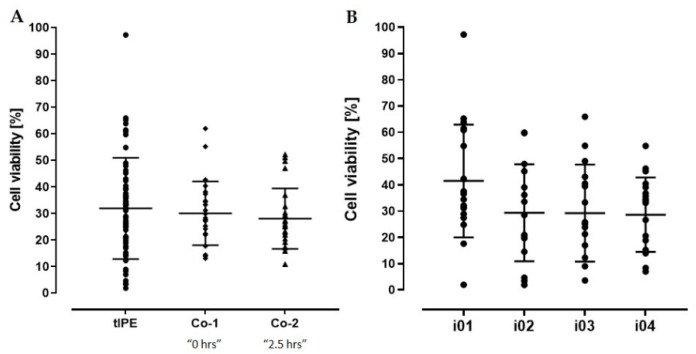
Cell viability of tIPE and non-transfected control cells. (**A**) There was no difference in the percentage of viable cells between tIPE and controls (tIPE: 31.92 ± 19.04%; Co-1: 30.062 ± 12.04%; Co-2: 28.02 ± 11.40%) (*p* > 0.999). (**B**) Individual analysis of the iridectomies i01–i04 showed that cell viability did not decrease during the time elapsed while processing one eye (i01: 41.46 ± 21.45%; i02: 29.35 ± 18.48%; i03: 29.23 ± 18.46%; i04: 28.64 ± 14.18%) (*p* = 0.2360).

*Cell viability.* The mean “time from donor death to cell isolation” for the eyes available for the ValRun was 7.35 ± 1.29 d (range 2–8 d). We assume that this factor decreased cell viability compared to biopsies harvested from patients and thus, we did not define an absolute percentage of viable cells as specification in the ValRun but the difference in viable cells in tIPE to control cells (Co-1 and Co-2) should not be significant. Since the controls were not mixed with plasmid DNA, electroporated, or transported, a comparable percentage of viable cells in tIPE and controls excludes a negative impact of the production procedure on cell viability. Figure 7 illustrates that the manipulations necessary for the manufacturing and transport of tIPE did not impact cell viability.

**Figure 8:**
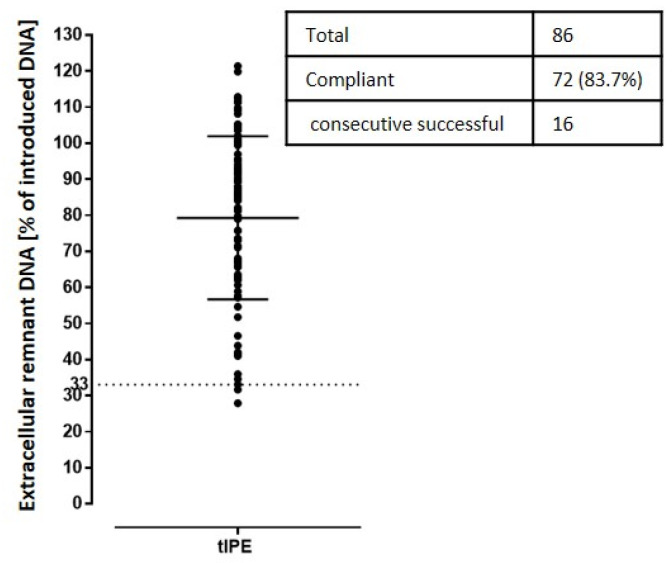
Extracellular remnant DNA. Remaining remnant extracellular DNA potentially contaminating tIPE, was measured in the centrifugation supernatant. Removal was successful in 72 out of 86 productions. The dashed line marks the limit for product release.

*Remnant extracellular DNA.* After transfection, remnant extracellular DNA was removed from tIPE by centrifugation with DNA remaining in the supernatant. The remnant extracellular DNA in the supernatant measured for 86 productions showed that the majority of extracellular DNA (80.32 ± 21.31%) was removed. Of these 86 productions, 14 were non-compliant, of which 13 were non-compliant due to a too high fluorescence to reliably calculate the free extracellular DNA concentration (invalid) and only one measurement was non-conforming (Figure 8).

**Figure 9:**
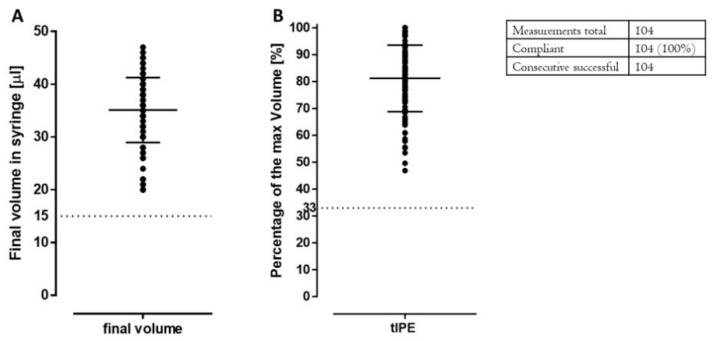
Volume of tIPE. (**A**) illustrates the volume of tIPE in microliter, and (**B**) shows the volume in percentage of the maximal volume. The dashed line marks the minimum volume for product release.

*tIPE volume.* After centrifugation, the cell pellet was resuspended in 50 µL BSS Plus and tIPE filled into the Hamilton syringe. However, the final volume in the syringe was less than 50 µL since an aliquot of 1000 cells was removed to analyze cell viability. Consequently, the final volume of tIPE was different for every batch. The specification and release criterion of 15 µL was defined by following consideration: Given the mean number of cells isolated from one biopsy (17,882 ± 11,725 cells), the necessary cell number of ≥5000 cells will be available in ≥15 µL tIPE. The volume was conforming in all productions, with a mean of 35.34 ± 6.0 µL (81.18 ± 12.37%) (Figure 9). The absence of air bubbles in the syringe was verified in all 104 productions.

**Figure 10:**
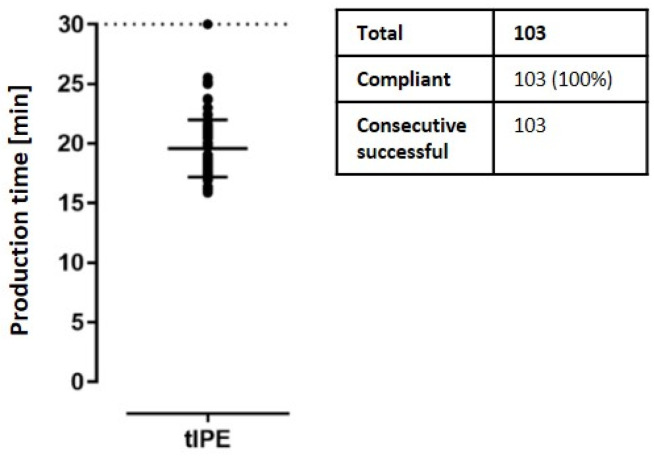
Production time. All recorded 103 productions were below the set 30 min time limit (dashed line).

*Production time.* Since the production time might affect cell viability, it was recorded. To standardize tIPE manufacture, an *a priori* time limit of 30 min per tIPE manufacture was set, which would allow for cell transplantation into the subretinal space of a patient to be accomplished within one hour. Documentation of the time for the recorded 103 tIPE productions was 19.59 ± 2.39 min (Figure 10). Additionally, we did not find a reduction in cell viability during the time that elapsed between the first and fourth tIPE manufacture from a single eye (Figure 7).

*Endotoxin level.* Theoretically, cell products can be contaminated by endotoxins, why we determined their level in the ValRun. Of 97 tIPE productions for which the endotoxin levels were analyzed, 90 (92.8%) had endotoxin levels below the minimum allowed with the last 13 consecutive productions being conforming. The 7 non-compliant tIPE productions were non-compliant due to invalid endotoxin measurements.

*Aseptic production and sterility of iris biopsies and tIPE.* Performance of manufacture at aseptic conditions is key to produce an ATMP in pharmacological quality. We tested environmental, biopsy and tIPE sterility for all productions.

- Environmental sterility. Even though four biopsies were taken and four tIPE manufactured from each donor’s eye, during the ValRun only one environmental control per eye was analyzed for environmental microbiological contamination. In the case of a positive result, all four tIPEs from the respective eye were considered non-compliant. Results showed that 20 of 26 analyses (76.9%) were sterile with the last 8 consecutive analyses conforming. Particle analysis performed by the Labguard^®^ system (Biomérieux, Petit Lancy, Switzerland) verified a GMP-conforming environment for 100 out of 104 productions (96.2%).
- Biopsy sterility. Analysis of the stroma from the iris biopsies showed that 96.2% (100) were sterile including the last 8 consecutive analyses.
- tIPE sterility. Analysis of the supernatant from the centrifugation after electroporation showed that 96.2% (100) of tIPE manufactured, including the last 8 consecutives, were sterile.

**Figure 11:**
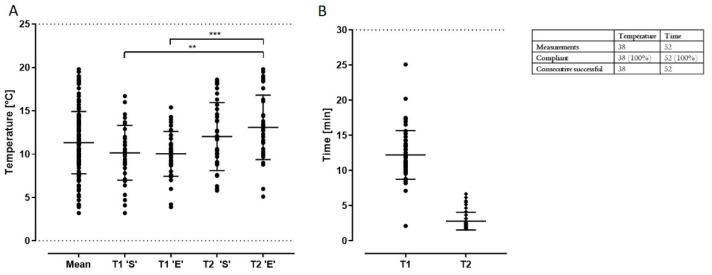
Transport validation. (**A**) The graph shows the temperature in the transport box for biopsies transported from the laboratory to the LTTC (T1) and tIPEs from the LTTC to the laboratory (T2). Note the temperature increase during transport remained within acceptable limits (T1′S′ vs. T2′E′: *p* = 0.0013, **; T1′E′ vs. T2′E′: *p* = 0.0007, ***). (**B**) The mean duration of both, T1 and T2 was within the accepted range of 30 min. Temperature was recorded for 38 and time for 52 transports of which 100% were conforming. S = start of transport; T = end of transport.

*Transport time & temperature.* The temperature was recorded when the biopsy or tIPE was placed into the transport box (T1′S′ for biopsies and T2′S′ for tIPEs) and when it was taken out of the transport box (T1′E′ for biopsies and T2′E′ for tIPEs). Even though the cooling packs thaw during the transport, the temperature for all 38 documented productions remained within the acceptable range of 4–25 °C with a mean temperature of 11.33 ± 3.59 °C. The duration of the 52 documented transports was within the limit of 30 min (T1: 12.22 ± 3.45 min; T2 2.80 ± 1.25 min) (Figure 11); the difference between T1 and T2 is detailed in the discussion.

*Overall compliance.* After analysis of the individual parameters, overall compliance was evaluated. We were able to demonstrate conformity for all release criteria, i.e., cell number, remnant extracellular DNA, production time, tIPE volume, endotoxin level, aseptic processing and sterility. We were also able to show that cell viability was not significantly reduced during production and successfully validated the transport protocol. Product batches with missing values were counted as invalid but were not included in the calculation of compliant productions. Eighty-eight of 104 tIPE manufactured were valid productions (84.6%) and 52 of these were compliant (59.1%) with eight consecutive compliant productions at the end of the series.

*Qualification Cliniporator^TM^*. The performance of the Cliniporator^TM^ was automatically recorded for every production and archived. The ValRun successfully qualified the performance of the new device with 100% reliability.

*Qualification personnel.* Of the three operators assigned to manufacture tIPE two (P1 and P3) became qualified for tIPE production during the ValRun; operator P2, who could not complete the number of necessary valid productions during the ValRun, has since been qualified. Product quality did not vary significantly between the three operators demonstrating robustness of the standardized process (Table 5).

## 4. Discussion

Here, the validation of the manufacture process and QC for batch release of the new ATMP tIPE has been described. The specific challenges included the following: setting the GMP requirements, standardization, assessment of its biological nature, short production times, potential alterations of the ATMP ex vivo (e.g., cell death), small sized autologous products whose amount is limited to clinical dose, irreversible cell labelling preventing cell characterization or purification, and the maintenance of a closed, GMP-compliant circuit from starting material collection to production, transport, and transplantation [9,34].

Existing QC methods for in-process cell analysis include magnetic and/or fluorescent cell sorting integrated into production platforms such as the CliniMACS^®^ system (Miltenyi, Bergisch Gladbach, Germany). However, this particular QC method requires sample processing with the loss of the analyzed cells and without providing a characterization of the complete batch. Other services and instruments for classical analytical methods of cell culture medium or lysed cells, e.g., ELISA, HPLC and PCR require from a few hours up to several days (e.g., Solvias, Kaiseraugst, Switzerland) without providing complete characterization. Recent approaches have been developed that provide more comprehensive testing such as multiplex ELISA (e.g., ThermoFisher Scientific, Waltham, MA, USA) [35,36]. Reversible labeling (REAlease^®^ MicroBead Technology, Miltenyi, Bergisch Gladbach, Germany), and microfluidics have been developed for damage-free cell sorting with a fluorescent label (PHC) or label-free using impedance cytometry (Zurich Instruments, Zurich, Switzerland). Collectively, these techniques require long time and often large samples and thus, are not suitable for ATMPs that have a limited number of cells and/or requiring transplantation within a short period of time, such as for tIPE. It is not recommended to culture the cells for later transplantation, as it has the potential to alter the cell’s phenotype as well as introduce harmful contaminants [37]. Other types of ATMPs like in vivo administered vector-gene constructs, such as LUXTURNA^®^, which is an adeno-associated virus (AAV) serotype 2- human RPE65 construct to deliver the *RPE65* gene to RPE cells allow comprehensive process validation and QC [38] using classical procedures since the construct does not degrade with time and can be produced in excess [39].

Since currently available methods are not appropriate for QC analyses for batch release of tIPE, according to the risk-based approach (RBA) suggested by Eudralex [11], we analyzed key intermediates in-process and relied on a comprehensive process validation, during which the whole product was used for analysis. Following the quality by design (QBD) approach [40] we defined the critical quality attributes (CQA) of tIPE, specifically, suspension in functional BSS Plus, cell number, product volume, method of administration, absence of biocontaminants, absence of endotoxins (<0.2 EU/mL), minimal remnant extracellular DNA, and stable cell viability throughout the time of tIPE manufacture. The identified critical process parameters (CPP) are homogenous suspension in BSS Plus, integral triple packaging, time of production, temperature and reliability of transport.

Since the treatment protocol for tIPE is designed to transplant the ATMP within approximately one hour after taking a biopsy from the patient, tIPE results of analyses for sterility will not be available before release. Thus, the cleaning procedure validation (equipment and environment) has been integrated within the comprehensive, extended ValRun, which included 104 productions, though commonly, one PQ for device qualification is sufficient and the qualification of personnel, validation of cleaning and manufacture processes is sufficient to be performed in three consecutive batches [11].

The validation process was started with the definition and analysis of limits for the identified CQAs and CPPs to determine training effects and to eliminate the latter from the final validation. Only productions performed after reaching a level of quality stably compliant, were integrated into the ValRun. In addition, the 104 productions from 26 donors addressed adequately the inherent variability of the starting material. The high number of productions and the inherent variability of the starting material also required the definition of benchmarks for successful validation and differentiation between the conformity of a release criterion, compliance of the production process as a whole and personnel qualification as described in Section 3.3.1. We were able to show conformity of all CQAs and CPPs including stable cell viability in >75% of productions. The cleaning validation was shown by environmental controls, which were negative in 76.9% and sterility analyses of starting material and final product were negative for 96.2% of biopsies and supernatant samples, which conform with the established parameters. In reanalyzing the process during the ValRun, the initial packaging of the biopsies was identified as a potential step responsible for contamination. Implementing additional disinfection measures improved the process and following 32 consecutive environmental controls were compliant. It has to be noted that the number of both, positive and negative environmental controls, is biased since the analysis was performed on each eye and not on each of the four biopsies from a single eye. To ensure sterility during delivery of tIPE from the manufacturing to the surgical site, a customized bioconfined container for transportation of tIPE was designed to be a simple, economical and commercially competitive product, manufactured using 3D injection printing [41]. The material was chosen to make it autoclavable and therefore reusable [42] with a disposable internal syringe holder. An enhanced version with a spring in which the syringe will be clamped to “close” the syringe during transport is in development and will further improve safety. The PQ of the Cliniporator^TM^ functioned without difficulty for all 104 ValRun productions. A successful manufacture process validation requiring ≥50% of compliant productions, including six consecutive compliant productions, was successfully accomplished. Finally, the ValRun qualified the operators successfully.

The ValRun was designed to mimic the proposed clinical trial protocol as closely as possible. The strengths of the study in regard to the proposed clinical trial were: (a) use of the same Standard Operating Procedures (SOP), specifically created for tIPE production, (b) iris biopsies taken from human eyes, (c) similar hygienic conditions for iris biopsy preparation, (d) similar transport settings. The differences from the proposed clinical trial and thus limitations of the ValRun were: (a) iridectomies were performed by a team member and not by the surgeon, (b) iris biopsies were cut through a larger corneal incision, which is different from surgical practice, (c) four biopsies were taken from one eye whereas for the proposed clinical trial one biopsy will be taken from the patient’s eye. The data presented here is derived from IPE cells isolated up to 8 days *post-mortem* from donor eyes, which may be not as vital as cells isolated from a patient’s biopsy. Our data shows that viability of IPE cells isolated from *post-mortem* donor eyes is 31.92 ± 19.04% whereas Lappas et al. determined viability of patient-derived IPE cells to be 75.45% [21]. For the proposed clinical trial, the results of sterility analyses will be available only after transplantation of tIPE, which poses a risk; however, this risk is acceptable since the percentage of contaminations was low during the ValRun, the customized biocontainer ensures a closed manufacture and transport circuit; additionally, post-operative fundoscopies allow identification of early signs of infection and initiation of appropriate treatment. Nevertheless, the prediction of the quality of autologous, personalized products by pre-clinical data, validation procedures and in-process controls is limited. A Japanese study demonstrated the advantage of comprehensive QC before transplantation: iPS-derived RPE cells should be transplanted to AMD patients and only the QC performed pre-transplantation allowed the discovery of potentially harmful aberrations in DNA copy number in one transplant [43]. However, for tIPE, transfection related side effects are minimal since the integration profile of *SB*-based modified cells have a negligible risk of integration into active genes and oncogenes [20] and no known tumorigenic activity [44]. The risk of toxic side effects from cell debris, the vector or the PEDF protein secreted by the cells are minimal since debris and remnant extracellular (vector and chromosomal) DNA are removed during tIPE manufacture and PEDF is ubiquitously expressed in the human body [45]. For safety reasons, the guarantee of successful removal of remnant extracellular DNA was given priority to the cell loss during centrifugation. Since the cells are transplanted into the eye, they are easily accessible and can be destroyed by laser ablation if necessary. The only remaining limit of the present process is the lack of a method to determine the efficacy of individual batches of tIPE before transplantation, since current methodologies to determine efficacy or transgene integration rate are not suitable for ATMP QC [46,47].

As aforementioned, transport settings were similar to those foreseen in the clinical trial. The distance from the laboratory and the surgery room, respectively, to the LTTC are similar, and though tIPE was not transplanted after production in the ValRun, it was similarly packed as it would have been in the regular clinical use. The team member responsible for the transport recorded the time from iridectomy to the LTTC and the time after production back to the laboratory. It has to be noted that before transferring the iris samples to the team in the LTTC, the carrier had to prepare the samples for entry to the platform, dress himself for entry and stopped the time after coming back to the locker room and the successful transfer; therefore, this time “T1” is longer than “T2” that purely recorded the time necessary for the way from the locker room to the laboratory. Nevertheless, the total procedure time (adding T1, T2 and the production time) remains far below the limit of 60 min (30 min for the transport plus 30 min for the production) with a mean procedure time of 34.59 min. Transport temperature was always in defined limits; nevertheless, during the scheduled clinical trial, a box with electronically controlled cooling elements will enhance standardization of temperature conditions.

To verify that the proposed treatment approach, namely taking an iris biopsy from a patient, isolating, transfecting and transplanting the cells subretinally in the same patient within one hour, is feasible, a study was carried out in rabbits that simulated the proposed protocol considering that isolating cells from the rabbit iris requires 75 min trypsinization whereas in humans, IPE cells can be scraped from the stroma without pretreatment. The transport from the animal surgery room to the laboratory, where cell isolation took place, is longer (~7 min) than the path from the clinic’s surgery room to the LTTC (~2 min). Isolated rabbit IPE cells were used to manufacture a cell product similar to tIPE following the protocol for the proposed human clinical trial. Subtracting the time for IPE cell trypsinization, the overall process from biopsy to transplantation was feasible in 60 min as scheduled for the clinical trial. After transplantation, rabbits were monitored for 7 d or 90 d and no adverse effect or organ abnormalities were observed. The weight loss in rabbit no. 4 was considered not related to tIPE since all other measured parameters were normal. We hypothesize that the lowered IOP at day 7 resulted from the breaching of the posterior chamber during the subretinal transplantation of tIPE.

In the ValRun, both individual parameters and the overall compliance were evaluated. The results show variability, which resulted from the unknown quality of the starting material, i.e., donor eye-derived IPE cells. We were able to demonstrate compliance for all release criteria (cell number, remnant extracellular DNA, production duration, tIPE volume, endotoxin level, aseptic processing and sterility of biologic material). We confirmed that cell viability is not significantly reduced during production and successfully validated the transport protocol. The ValRun confirmed an aseptic manufacture though the design of environmental controls was influenced by the ValRun set-up, which had to make compromises, since we performed one control per eye from which 6 biopsies had been collected, while in the real surgical setting every production will be controlled. Product batches with missing values were counted as non-applicable and not included in the calculation of compliant productions. Overall, 52 tIPE manufactured were compliant with all parameters with 8 consecutive compliant productions at the end of the series. A set of analyses was designed to be performed rapidly during tIPE manufacture and to be transferable to analyze tIPE manufactured from a patient’s biopsy during the proposed clinical trial. The absence of complex techniques permits implementation at other tIPE manufacture centers as well as being adaptable to comparable ATMPs.

## 5. Conclusions

Advanced therapies offer promise for millions of patients but manufacture of GMP-compliant ATMPs is challenging [9,13,40] often requiring the development of novel methodologies to simplify the workflow in closed and automated systems. For the validation of tIPE, the protocol comprises an in vivo ValRun that is feasible for any novel cell therapy displaying strong constraints in cell number and production time. The ex vivo ValRun using human donor tissue successfully validated the manufacture, qualified devices, and personnel for application to future tIPE manufacture and the implementation of a “simplified” QC protocol for clinical use. The standardized procedure allows the transfer of tIPE manufacturing to other clinical centers and comparable ATMPs for the broad application of this and similar therapies. The first in-human clinical application of tIPE for the treatment of nvAMD is planned for 2023 at one clinical center followed by a multi-centric phase II trial.

## Figures and Tables

**Table 1:** Welfare of treated rabbits. Rabbits nos. 1 and 2 were monitored for 90 d while nos. 3 and 4 were sacrificed at 7 d. The weight was determined weekly. Behavior, food and water intake, condition of fur (groomed and shiny), eyes (clear, no redness, swelling or discharge), and frequency of blinking (qualitatively) evaluated weekly and any anomalies were noted. The shown Δ weight refers to the change from day 0 to 7. N = normal.

Animal No.	No. Examinations	Δ Weight (%)	Behavior	Food/Water Intake	Fur	Blinking	Eyes
1	11	0	N	N	N	N	N
2	11	−2.6	N	N	N	N	N
3	2	−2.2	N	N	N	N	N
4	2	−11.1	N	N	N	N	N

**Table 2:** Fundus and IOP of treated rabbits. The fundus of treated rabbits was examined in both eyes and found to be normal in all rabbits at all time points. IOP was measured pre- and post-treatment in the treated and contralateral eye; a small decrease in IOP was noted during the first week that recovered without further intervention. Comparison between both eyes in animal 1 and in animal 2 revealed no statistical differences (paired *t*-test).

Animal No.	Fundus Morphology	IOP Treated Eye (mm/Hg)	IOP Contralateral Eye (mm/Hg)	*p*-Value
1	Normal	13 (day 0)	14 (day 0)	*p* > 0.999
		8 (day 7)	10 (day 7)	
		7 (day 90)	8 (day 90)	
2	Normal	14 (day 0)	12 (day 0)	*p* = 0.580
		6 (day 7)	8 (day 7)	
		9 (day 90)	10 (day 90)	

**Table 3:** Donor characteristics of eyes used for training and validation. m = male; f = female.

	Total (Mean ± SD)	Training (Mean ± SD)	ValRun (Mean ± SD)
**No. of donors**	28	11	17
**No. of eyes**	42	16	26
**No. of productions**	143	39	104
**Age of donors** (years)	81.18 ± 11.73	78.09 ± 15.46	83.65 ± 8.93
**Sex of donors** (m/f)	12/16	3/8	8/9
**Time of death to cell isolation** (d)	7.24 ± 1.23	7.45 ± 0.82	7.35 ± 1.29

**Table 4:** QC and specifications defined for tIPE manufacturing during validation. List of controls performed during validation as shown in Figure 4 (compared to controls planned to be performed in the clinical trial), the methods used and their importance for batch release. Generally, all used consumables including the sterile single use electroporation cuvettes were verified for package integrity, expiration date, and lot numbers were documented. N.A. = not applicable.

Release Criterion/Phase	Method for Its Determination	Specification	Action (In Case of a Result OOS)	
			Validation Run	Clinical Trial
**Starting material** (iridectomy)	Visual control: no saliencies	0	Stop production	Stop production
**Cliniporator^TM^**	Self-test	“Passed“	Repetition of self-test	Repetition of self-test
			2nd self-test OOS: stop production	2nd self-test OOS: stop production
**Plasmid** **mixture**	Visual control: vial intact & frozen	“Yes”	New vial	New vial
			No conforming vial available: continue production	No conforming vial available: stop production
**3P.14 buffer**	Visual control: vial intact & frozen	“Yes”	New vial	New vial
			No conforming vial available: continue production	No conforming vial available: stop production
**Transport**	Visual control: tape intact	“Yes”	Continue production; record time & temperature	Stop production
**Starting material** (stroma)	Eur. Ph. Method 11.0, 2.6.: microbiology (stroma)	0 CFU	N.A.	Antibiotic regimen; not critical for batch release
**Homogenization**	Visual control: homogenous suspension	“Yes”	Repeat resuspension	Repeat resuspension
**Volume**	Visual control: 15 µL	<15 µL	Add 3P.14 buffer up to 15 µL	Add 3P.14 buffer up to 15 µL
**Electroporation**	Normal performance	No error message	Continue production	Stop production
**Washing** (centrifugation)	Divide supernatant into 3 parts:			
tIPE	Eur. Ph. Method 11.0, 2.6.: microbiology	0 CFU	N.A.	Antibiotic regimen; not critical for batch release
Bacterial endotoxin	EndoSafe^®^, colorimetric	<0.2 EU/mL	Continue production	Stop production
Remnant extracellular DNA	Qubit dsDNA assay, fluorimetric	≥33%	N.A.	N.A. (not measured)
**Cell number**	Neubauer chamber: counting	≥5000 cells	Continue production	Continue production; open a deviation
**Cell viability**	Percentage viable cells	tIPE ≈ Co-1/2	N.A.	N.A. (not measured)
**Filling syringe**	Visual control: absence of bubbles	<1	Repeat filling up to 3 times	Repeat filling up to 3 times
	Visual control: volume	≥15 µL/33%	3rd try failed: continue production	3rd try failed: stop production
**Duration**	Timer: time	≤30 min	Continue production	Continue production; open a deviation
**tIPE labelling**	Completeness and readability	“Yes”	Correct labelling	Correct labelling
**Air samples, settle plates, contact plates, glove prints**	Eur. Ph. Method 11.0, 2.6.: microbiology	0 CFU	N.A.	Antibiotic regimen; not critical for batch release.
**Particle control**	Logiview and Labguard^®^-controlled: inorganic particles	Class A 0.5 µm/m^3^: <3520 5 µm/m^3^: <29 Class C 0.5 µm/m^3^: <3.5 Mio 5 µm/m^3^: <29,300	Continue production	Stop production
**Dossier**	3-fold control: completeness, readability, correctness	“Yes”	Correction	Correction
			No correction possible: documentation of non-compliant production	No correction possible: no release

**Table 5:** Personnel qualification (P1–P3). Three team members were qualified for tIPE manufacture. The parameters necessary to be conforming for qualification are shown in the first column: number of total, valid, compliant, and consecutive (at the end of the series) compliant productions, number of cells isolated from biopsies, cell viability, remnant extracellular DNA, time of tIPE manufacture, volume of tIPE in percentage of maximal volume, endotoxin level, sterility of starting material, i.e., iris biopsies. P1 and P3 were successfully qualified after >20 valid productions, in which the mean of the individual parameters was conforming and ≥50% of the batches were compliant in all parameters. P1 accomplished 35 valid productions of which 23 were compliant. P3 performed 34 valid productions of which 19 were compliant. By the end of the ValRun, P2 achieved 19 valid productions of which 10 were compliant. n.a. = not applicable.

	P1			P2			P3		
	N (%)	Value (Mean ± SD)	Conforming (%)	N (%)	Value (Mean ± SD)	Conforming (%)	N (%)	Value (Mean ± SD)	Conforming (%)
**Productions**									
Total	40			24			40		
Valid	35			19			34		
Compliant	23 (65.7)			10 (52.6)			19 (55.9)		
Consecutive compliant	7			0			5		
**Cells**									
No.	40	18,060 ± 9291	38 (95.0)	24	17,448 ± 15,824	23 (95.8)	40	18,238 ± 10,948	39 (97.5)
Viability (%)	28	31.76 ± 20.65	n.a.	12	28.9 ± 18.7	n.a.	28	33.4 ± 18.02	n.a.
**Remnant extracellular DNA** (%)	33	82.74 ± 22.98	26 (78.8)	20	74.8 ± 24.2	15 (75.0)	34	80.9 ± 19.3	32 (94.1)
**Time** (min)	39	19.1 ± 2.15	39 (100)	24	19.88 ± 2.21	24 (100)	40	20.12 ± 2.78	40 (100)
**tIPE volume** (µL)	40	79.7 ± 11.2	40 (100)	24	83.4 ± 12.1	24 (100)	40	81.3 ± 13.7	40 (100)
**Endotoxins**	40	n.a.	39 (97.5)	24	n.a.	24 (100)	39	n.a.	33 (84.6)
**Iridectomy sterility**	40	n.a.	38 (95.0)	24	n.a.	23 (95.8)	40	n.a.	39 (97.5)

## Data Availability

Data supporting reported results can be found on the Zenodo repository https://doi.org/10.5281/zenodo.7249033; 25 October 2022.
